# 7^th^ Brazilian Guideline of Arterial Hypertension: Chapter 12 - Secondary Arterial Hypertension

**DOI:** 10.5935/abc.20160162

**Published:** 2016-09

**Authors:** MVB Malachias, LA Bortolotto, LF Drager, FAO Borelli, LAD Lotaif, LC Martins

## Introduction

Secondary AH has a prevalence of 3-5%. The treatment of the cause can cure AH or improve BP control. Chart 1 shows the situations in which secondary causes of AH should be investigated.

**Chart 1:** Major causes of secondary AH, signs and diagnostic screening

Clinical findings	Diagnostic suspicion	Additional studies
Snoring, daytime sleepiness, MS	OSAHS	Berlin questionnaire, polysomnography or home respiratory polygraphy with at least 5 episodes of apnea and/or hypopnea per sleep hour
RAH and/or hypopotassemia (not necessary) and/or adrenal nodule	Primary hyperaldosteronism (adrenal hyperplasia or adenoma)	Measurements of Aldo (>15 ng/dL) and plasma renin activity/concentration; Aldo/renin > 30. Confirmatory tests (furosemide and captopril). Imaging tests: thin-sliced CT or MRI
Edema, anorexia, fatigue, high creatinine and urea, urine sediment changes	Parenchymal kidney disease	Urinalysis, GFR calculation, renal US, search for albuminuria/proteinuria
Abdominal murmur, sudden APE, renal function changes due to drugs that block the RAAS	Renovascular disease	Renal Doppler US and/or renogram, angiography via MRI or CT, renal arteriography
Absent or decreased femoral pulses, decreased BP in the lower limbs, chest X ray changes	Coarctation of the aorta	Echocardiogram and/or chest angiography via CT
Weight gain, decreased libido, fatigue, hirsutism, amenorrhea, moon face, “buffalo hump”, purple striae, central obesity, hypopotassemia	Cushing’s syndrome (hyperplasia, adenoma and excessive production of ACTH)	Salivary cortisol, 24-h urine free cortisol and suppression test: morning cortisol (8h) and 8 hours after administration of dexamethasone (1 mg) at 24h. MRI
Paroxysmal AH with headache, sweating and palpitations	Pheochromocytoma	Free plasma metanephrines, plasma catecholamines and urine metanephrines. CT and MRI
Fatigue, weight gain, hair loss, DAH, muscle weakness	Hypothyroidism	TSH and free T4
Increased sensitivity to heat, weight loss, palpitations, exophthalmos, hyperthermia, hyperreflexia, tremors, tachycardia	Hyperthyroidism	TSH and free T4
Renal lithiasis, osteoporosis, depression, lethargy, muscle weakness or spasms, thirst, polyuria	Hyperparathyroidism (hyperplasia or adenoma)	Plasma calcium and PTH
Headache, fatigue, visual disorders, enlarged hands, feet and tongue	Acromegaly	Baseline IGF-1 and GH and during oral glucose tolerance test

OSAHS: obstructive sleep apnea-hypopnea syndrome; Aldo: aldosterone; RAH: resistant arterial hypertension; GFR: glomerular filtration ratio; APE: acute pulmonary edema; RAAS: renin-angiotensin-aldosterone system; CT: computed tomography; ACTH: adrenocorticotropin; TSH: thyroid stimulating hormone; PTH: parathormone; IGF-1: insulin-like growth factor type 1; GH: growth hormone.

### Chronic kidney disease

Chronic kidney disease is defined by a GFR < 60 mL/min or abnormal findings in urinalysis and/or kidney morphology for 3 months.^1^ As CKD advances, AH increases progressively, affecting 90% of stage 5 patients.^2^

All patients with AH should have plasma creatinine measured, their GFR calculated and urinalysis performed to screen for CKD.^3^ (GR: I; LE: A). Additional investigation includes renal US for all.^3^ Other exams (albuminuria, CT, MRI) can be necessary. Kidney biopsy is indisputably indicated in the presence of rapid decline in glomerular filtration or proteinuria > 3.5 g/g of urine creatinine.^4^ Arterial hypertension accelerates the progression of CKD^5^ and BP reduction attenuates CKD course.^6^ The treatment goals and most indicated medications for BP control in patients with CKD are described in Chapter 8. For CKD patients on dialysis, BP reduction decreases mortality,^7^ and loop DIUs are indicated in the presence of residual renal function, as well as ultrafiltration, in selected cases.^8^

### Renovascular hypertension

Renovascular hypertension (RVAH) is secondary to partial or total, uni- or bilateral stenosis of the renal artery or of one of its branches, triggered and maintained by renal tissue ischemia. The RVAH prevalence is 5% of hypertensive patients.^9^ Its major cause is atherosclerosis (90%), followed by renal artery fibromuscular dysplasia,^10^ Takayasu's arteritis being the less frequent.^9^ Regardless of its cause, it is an important determinant of CV morbidity and mortality.^10^

The diagnosis and assessment of the extent of involvement with TOD are essential for the choice of treatment. A cost-effective investigation requires proper selection of candidates, and anatomical and functional assessment of the stenosis, in addition to methods to correct the anatomical and functional defect.^11^ Charts 2 and 3^12-14^ list the main steps.

**Chart 2:** ACC/AHA recommendations for renal artery stenosis search during coronary angiography

Clinical characteristics	Level of evidence
Beginning of hypertension < 30 years	B
Beginning of severe hypertension > 55 years	B
Accelerated/malignant hypertension	C
Resistant hypertension	C
Uremia or renal function worsening after use of ACEI or ARB (> 30% drop in glomerular filtration)	B
Atrophic kidney of unknown cause or size discrepancy between the two kidneys > 1.5 cm	B
Unexpected sudden pulmonary edema (mainly in uremic patients)	B

**Chart 3:** Clinical indicators of probable renovascular hypertension

Probability	Clinical characteristics
Low (0.2%)	Uncomplicated borderline or mild/moderate AH
Intermediate (5-15%)	Severe or resistant AH Recent AH < 30 years or > 50 years Presence of abdominal murmur Asymmetry of radial or carotid pulses Moderate AH associated with smoking or atherosclerosis in another site (coronary or carotid) Undefined renal functional deficit Exaggerated BP response to ACEIs
High (25%)	Severe or resistant AH with progressive renal failure Accelerated or malignant AH Sudden APE ACEI-induced creatinine increase Asymmetry of renal size or function

The indication for the therapeutic option should consider the etiology and clinical conditions associated with renal artery stenosis, such as AH, ischemic nephropathy and accelerated CVD. Evidence of benefit of the percutaneous or surgical mechanical treatment is restricted to situations, such as progressive renal function loss, APE and difficulty to control BP, that cause irreversible TOD.^15^ Regarding patients with RVAH due to fibromuscular dysplasia, 82-100% of them have BP control, and 10%, restenosis.^11^ (GR: IIa; LE: B). Regarding atherosclerotic RVAH without complications, three randomized studies have shown no benefit of stent implantation as compared to optimized clinical treatment in BP control, kidney disease progression, and occurrence of clinical events and mortality.^16-18^ For patients with atherosclerotic renal artery stenosis and controlled BP with clinical treatment, without heart complications and stable kidney function for 6-12 months, the mechanical intervention is not recommended, clinical treatment being the first option. (GR: II; LE: B).

**Figure 1:**
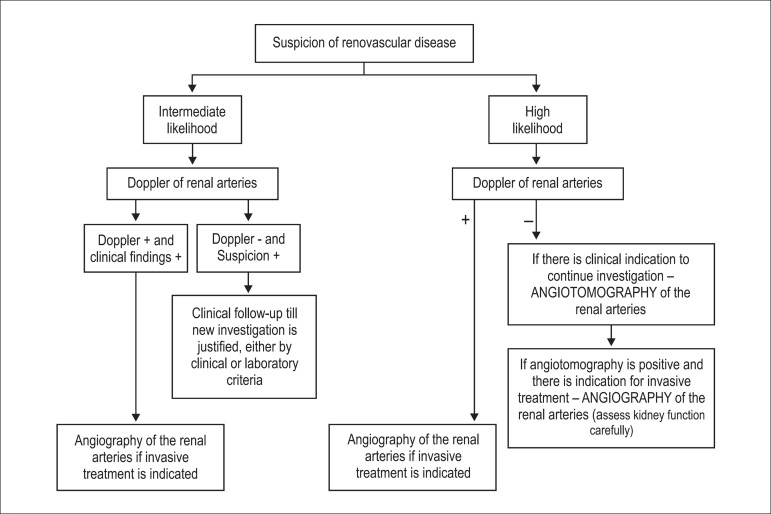
Flowchart for the investigation of patients suspected of having renal artery stenosis.

Figure 1 shows a flowchart for the assessment of patients suspected of having renal artery stenosis.

### Obstructive sleep apnea-hypopnea syndrome

Obstructive sleep apnea-hypopnea syndrome is characterized by recurring upper airway obstructions during sleep, causing reductions in intrathoracic pressure, intermittent hypoxia and sleep fragmentation.^19^ There is evidence that OSAHS is related to the development of AH regardless of obesity.^20,21^ The prevalence of OSAHS in patients with AH is 30-56%,^22,23^ reaching 64-83% in those with resistant AH (RAH).^24,25^ OSAHS contributes to TOD^26^ and acceleration of atherosclerosis in hypertensives.^27^

The risk factors for OSAHS are age, male sex, obesity and MS. The Berlin questionnaire^28^ can be used to screen for OSAHS,^23^ but does not seem useful in patients with RAH.^29^ Changes in the physiological BP decrease during nocturnal sleep can indicate the presence of OSAHS.^30^ Polysomnography or home respiratory polygraphy confirms the diagnosis with the finding of at least five episodes of apnea and/or hypopnea per hour of sleep (apnea-hypopnea index - AHI), and an AHI ≥15 events/hour seems to have a higher impact on AH.^31^

The treatment of choice for moderate or severe OSAHS is the use of continuous positive airway pressure (CPAP) during sleep.^31^ Meta-analyses have shown a small effect of CPAP in reducing BP, but they have limitations because they included studies on individuals with normal BP and controlled hypertensives.^32-34^ Most randomized studies^35-38^ on patients with OSAHS and RAH have shown more significant reductions in BP than those of patients with non-resistant AH. Body weight loss in combination with CPAP has resulted in greater BP reduction than each isolated intervention in obese individuals with OSAHS.^39^ Mandibular advancement with mobile orthodontic devices for mild to moderate OSAHS can also reduce BP,^34^ but further studies are necessary.^34^ Although several antihypertensive classes have been tested,^40^ there is no definitive conclusion about the best drug for hypertensives with OSAHS.^40,41^

### Primary hyperaldosteronism

Primary hyperaldosteronism (PHA) is a clinical condition characterized by excessive, inappropriate and autonomous production of aldosterone^42^ (Aldo), caused by bilateral adrenal hyperplasia or unilateral Aldo producing adenoma (APA), and, more rarely, unilateral adrenal hyperplasia, adrenal carcinoma or genetic origin (monogenic or chimeric gene). The prevalence of PHA in hypertensives is 3-22%, being higher in stage 3 and/or resistant hypertensives.^43^

Primary hyperaldosteronism is suspected when AH is associated with: spontaneous or DIU-induced hypokalemia; adrenal incidentaloma; RAH; family history of AH or CbVD before the age of 40 years; and MS. The prevalence of hypokalemia in PHA is 9-37%.^43^

**Figure 2:**
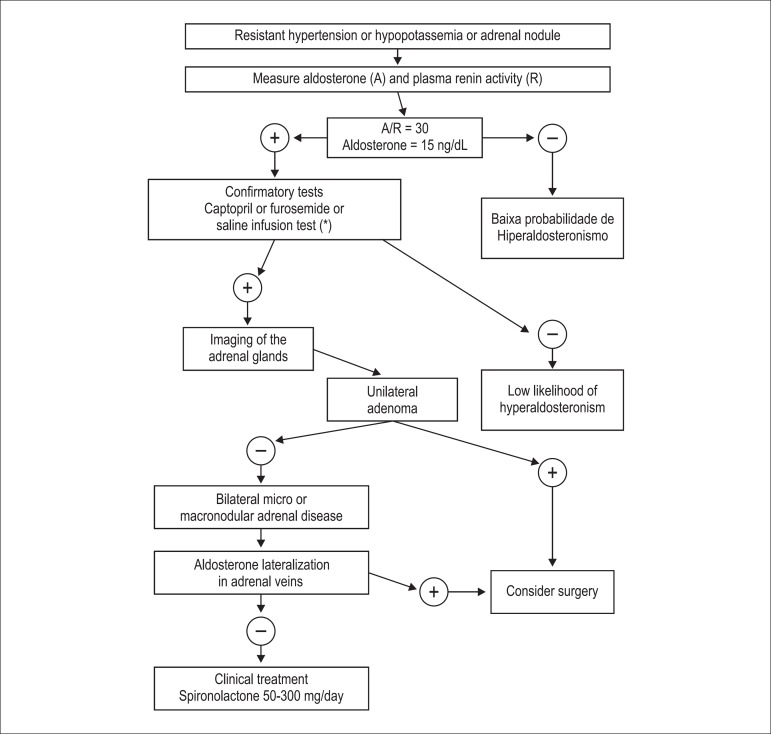
Flowchart for primary hyperaldosteronism screening, diagnostic confirmation and treatment. *The furosemide and captopril tests have higher diagnostic accuracy than the saline infusion test.

Figure 2 shows the flowchart for screening, diagnostic confirmation and treatment of PHA.

Laboratory tests do not require suspension of antihypertensive agents, except for spironolactone for 4-6 weeks.^43^ Suppressed plasma renin activity (PRA) and Aldo > 15 ng/dL, with an Aldo/PRA ratio > 30, indicate the diagnosis of PHA. Confirmatory testing is recommended when Aldo > 15 ng/dL and < 25 ng/dL, with an Aldo/PRA ratio > 30 and < 100. The furosemide and captopril tests have higher diagnostic accuracy than the saline infusion test.^44^ In the furosemide upright test, the patient should remain lying down for at least 30 minutes, then receive 40 mg of furosemide (IV), and renin should be measured after 2 hours of walking. The test is positive if PRA < 2 ng/mL/h. In the captopril challenge test, 50 mg of captopril are administered orally after the patient remained seated or in the upright position for at least 1 hour. Renin and Aldo should be measured at the times 0, 60 and 120 minutes. The test is positive if there is no drop > 30% in plasma Aldo or if it remains > 12 ng/dL. In the saline infusion test, 2 liters of 0.9% saline are administered (IV) in 4 hours. The Aldo measurement will be ≥ 5 ng/dL.

For APA or hyperplasia to be detected, thin-sliced CT or MRI of the adrenal glands is indicated.^43^ Catheterization of the adrenal veins is indicated when, on CT, the adrenal glands are normal, have bilateral abnormalities (thickening or micronodules) or a unilateral lesion in patients > 40 years.^44^ The dexamethasone suppression test is indicated to investigate PHA suppressible with glucocorticoid in patients with PHA and AH beginning before the age of 40 years.^44^

Laparoscopic surgery is indicated in APA,^43^ preferably with previous treatment with spironolactone up to 3-4 weeks.^45^ Clinical treatment of hyperplasia requires spironolactone, 50-300 mg/day, if well tolerated.^45^ Cure of AH with surgery is observed in 35-60% of the patients.^42,45^

### Pheochromocytomas

Pheochromocytomas (PHEO) are tumors of chromaffin cells of the sympathetic adrenomedullary axis that produce catecholamines.^46^ Of PHEOs, 10% to 15% are extraadrenal (paragangliomas), 10% are bilateral, and 10% are malignant.^47^ Familial forms have the dominant autosomal trait or are part of syndromes with known gene mutations.^47^

Presence of persistent or paroxysmal AH (50%), paroxysmal headache, excessive sweating and palpitations (classic triad)^46^ is indicative of the disease, and concomitance of the classic triad with HC has sensitivity of 89% and specificity of 67% for the PHEO diagnosis.^46^

Laboratory diagnosis is based on the measurement of catecholamines and their metabolites in blood and urine. Free plasma metanephrine has the highest sensitivity and specificity,^48^ but because of its higher cost, urine metanephrine isolated or associated with plasma catecholamines is indicated in cases of high likelihood.^48^ The measurement of urine vanillylmandelic acid has good specificity, but the lowest sensitivity of all methods, being indicated only when the other tests are not available.^48^ If the diagnosis is not certain, clonidine suppression test is indicated in hypertensives, and glucagon stimulation test, in individuals with normal BP levels.^47^

The imaging tests to locate adrenal tumors are CT and MRI, with sensitivity of 89% and 98%, respectively.^49^ The MRI is superior to identify paragangliomas. MIBG whole body scan is useful in extraadrenal, bilateral PHEOs, and metastases and relapses.^50^ Octreoscan, bone scan and positron-emission CT can be indicated when the localizing exams cited are negative or when investigating malignancy.^51^

The preferential treatment is surgery, whose preoperative preparation should include alpha1-blockers (doxazosin or prazosin) and appropriate hydration for at least 2 weeks before surgery.^52^ The chronic pharmacological treatment includes alpha1-blockers, BBs (only after beginning alpha1-blockers, in the presence of symptomatic tachycardia), CCBs, ACEIs and central action agonists.^52^ The paroxysmal HC of PHEO is a HE, and should be treated with SNP or injectable phentolamine and volume replacement, if necessary.^46^

Total and early removal of the neoplasm usually determines total remission of symptoms and cure of AH.^47,49^ For malignant PHEOs with unresectable metastases, the following are indicated: chemotherapy, embolization, radiotherapy, and, if possible, ablation with MIBG-131.^47^ Clinical, biochemical and radiological follow-up of the patients is essential to detect recurrences or metastases, in the malignant form, and other tumor in familial syndromes.

### Other endocrine causes

#### Hypothyroidism

In hypothyroidism, AH occurs in 20% of hypothyroid patients.^53^ The diagnosis is established by finding high TSH levels and gradual decrease in free T4. The most common clinical findings are weight gain, hair loss and muscle weakness. The treatment is initiated with thyroid hormone replacement,^53^ and, if AH persists, antihypertensive drugs are indicated. (GR: II; LE: C).

#### Hyperthyroidism

In hyperthyroidism, AH is a frequent finding in hyperthyroidism, and the clinical presentation mimics hyperadrenergic findings. The main symptoms are palpitation, tremor, fatigue, increased sensitivity to heat, hyperactivity, weight loss and emotional lability.^54^ The most important signs are exophthalmos, hyperthermia, hyperreflexia and humid skin.^54^ The diagnosis is confirmed by low TSH levels and high free T4 levels. The treatment usually normalizes BP. Beta-blockers are the first choice to control the adrenergic symptoms. (GR: IIb; LE: C).

#### Hyperparathyroidism

In hyperparathyroidism, there is excessive secretion of parathormone (PTH) by the parathyroid glands, with consequent hypercalcemia and hypophosphatemia.^55^ It can be caused by an adenoma or hyperplasia of the parathyroid glands. Secondary hyperparathyroidism results from a situation that induces hypocalcemia, CKD being the major cause. The most common symptoms are depression, thirst, polyuria, renal lithiasis, osteoporosis, lethargy, muscle weakness, muscle spasms, and renal function reduction. Arterial hypertension is present in up to 75% of the patients, and can be resistant.^43^ The diagnosis is established with plasma calcium and PTH measurement. Surgical correction of hyperparathyroidism can cure or reduce BP in hypertensives.^56^

#### Cushing's syndrome

Cushing's syndrome (CS) is a disorder caused by excessive cortisol levels associated with a deficiency in the control mechanism of the hypothalamus-hypophysis-adrenal axis and of the cortisol secretion circadian rhythm.^57^ It can result from adrenal tumors with autonomous cortisol production (benign or malignant adenoma), adrenal hyperplasia, excessive adrenocorticotropin (ACTH) production, or ectopic tumor.^57^ The prevalence of AH in CS is 80% in adults and 47% in children.^57^ The major signs and symptoms are decreased libido, central obesity, moon face, striae, muscle weakness, and hirsutism.^58^ The confirmatory tests are: 24-hour urine free cortisol; nocturnal salivary cortisol; dexamethasone suppression test; dexamethasone combined with corticotropin-releasing hormone test; and ACTH measurement.^58^ Pituitary MRI shows an adenoma in 35% to 60% of patients.^58^ Surgical removal of the tumor can cure AH, but 30% of the patients maintain SAH, and 25%, DAH.^59^ The AH duration before surgery correlates with postoperative AH persistence.^59^ Thiazides and furosemide should be avoided, because they can worsen hypokalemia, ACEIs and ARBs being recommended.^59^

#### Acromegaly

Acromegaly is usually caused by a pituitary adenoma that secrets growth hormone (GH) and insulin-like growth factor type 1 (IGF-1). It manifests as progressive excessive growth of the hands, feet and facial bones, increased interdental spacing, mandibular prognathism, macroglossia, excessive sweating, and respiratory, CV, metabolic-endocrine and skeletal-muscle changes.^60^ In acromegaly, AH has a 35% prevalence, and contributes to increase the disease's morbidity and mortality. Acromegalic cardiomyopathy contributes to raise BP, and can be aggravated by the coexistence of AH. The treatment of acromegaly reduces BP in parallel with GH reduction.^60^

#### Coarctation of the aorta

Coarctation of the aorta is the aortic constriction close to the ductus arteriosus or ligament, found mainly in children and young adults. Clinical suspicion is based on symptoms (epistaxis, headache and weakness of the legs on exertion or manifestations of HF, angina, aorta dissection or intracerebral hemorrhage) and physical exam (upper limb AH, with SBP at least 10 mm Hg greater in the brachial artery than in the popliteal artery; pulse absence or decrease in lower limbs; interscapular and thoracic systolic murmur).^61-63^

The imaging exams include: chest X ray (thoracic aorta with pre- and post-stenosis dilations, costal corrosion); echocardiogram (posterior protrusion, expanded isthmus, transverse aortic arch, and high velocity continuous jet in the coarctation site); angiography with MRI (details of coarctation and intercostal vessels). The MRI is the best method for assessment and post-intervention follow-up in young individuals, and does not require preoperative angiography. Invasive angiography is indicated when other imaging methods do not provide visualization of the coarctation, and to older individuals who can have CAD. The definition of significant coarctation requires pre- and post-coarctation pressure gradient > 20 mm Hg.^62^

Patients who do not undergo surgery have a higher incidence of CV events. The treatment is always interventional: endovascular procedure (younger individuals or children) or surgery (hypoplasia of the aortic arch and/or need for coarctation resection). The BP response to interventional treatment depends on the duration of AH prior to surgery and the patient's age. The cure of AH occurs in up to 50% of patients, but AH can reoccur later, especially if the intervention is performed at advanced age. The drugs of choice for both the preoperative period and residual AH after surgery are BBs and ACEIs.

### Drug-induced AH

Chart 4 shows the medicines and licit and illicit drugs related to AH development or worsening.

**Chart 4:** Medicines and illicit and licit drugs related to AH development or worsening

Drug class	Effect on BP and frequency	Suggested action
**Immunosuppressants** Cyclosporine, tacrolimus	Intense and frequent	ACEI and CCB (nifedipine/amlodipine). Adjust serum level. Reassess options
**Anti-inflammatory agents** Glucocorticoid Non-steroids (1 and 2 cyclo-oxygenase inhibitors)	Variable and frequent Occasional, very relevant with continuous use	Salt restriction, DIUs, decrease dose Observe renal function, use for a short period
**Anorexigenic/satiety drugs** Diethylpropion and others Sibutramine Vasoconstrictors, including ergot derivatives	Intense and frequent Intermediate, little relevance Variable, transient	Suspension or dose reduction Assess BP reduction with weight loss Use for a determined short period
**Hormones** Human erythropoietin Oral contraceptives Estrogen-replacement therapy (conjugated estrogens and estradiol) GH (adults)	Variable and frequent Variable, prevalence of up to 5% Variable Variable, dose-dependent	Assess hematocrit and dose weekly Assess method replacement with an expert Assess risk and cost-benefit Suspension
**Antidepressant drugs** Monoamine-oxidase inhibitors Tricyclics	Intense, infrequent Variable and frequent	Approach as adrenergic crisis Approach as adrenergic crisis
**Illicit drugs and alcohol** Amphetamine, cocaine and derivatives Alcohol	Acute, intense effect Dose-dependent Variable and dose-dependent Very prevalent	Approach as adrenergic crisis See non-pharmacological treatment
